# Effectiveness of intervention for aromatase inhibitor-associated musculoskeletal symptoms: A protocol for systematic review and meta-analysis

**DOI:** 10.1097/MD.0000000000028982

**Published:** 2022-03-11

**Authors:** Hui Meng, Yuhan Chen, Mingwei Yu, Xiaomin Wang

**Affiliations:** a Beijing University of Chinese Medicine, North of the Third Ring, Chaoyang District, Beijing, PR China; b Beijing Traditional Chinese Medicine Hospital, Capital Medical University, Dongcheng District, Beijing, PR China.

**Keywords:** aromatase inhibitor, bone loss, breast cancer, Chinese herbal decoction, systematic review

## Abstract

**Background:**

Aromatase inhibitor-associated musculoskeletal symptoms (AIMSS) are among the most common prominent side effects in patients using aromatase inhibitors (AIs) for breast cancer. Muscle and joint pain, morning stiffness, arthritis, and bone loss are common clinical symptoms in individuals. Traditional Chinese medicine (TCM) has been demonstrated to be useful in the treatment of AIMSS in previous investigations, although the sample sizes were limited, and systematic reviews were inadequate. The effectiveness and safety of TCM in the treatment of AIMSS will be investigated in this study.

**Methods:**

Randomized controlled trials from January 2010 to October 2021 were limited to English or Chinese. We searched PubMed, EMBASE, Cochrane Library, Web of Science, Medline, China Biomedical Database (CBM), China National Knowledge Infrastructure (CNKI), Wanfang database, and the VIP database. Two researchers reviewed the literature and retrieved the data independently. Review Manager V5.3.was used to conduct the statistical analysis.

**Results:**

This systematic review and meta-analysis presents the most recent data on the use of TCM to treat AIMSS and offers a scientifically sound foundation for therapeutic practice. Upon completion, the findings will be submitted to a peer-reviewed journal.

**Ethics and dissemination:**

As the systematic review protocol did not involve human subjects, ethical approval was not required.

**PROSPERO registration number:**

CRD42020192553.

## 1 Introduction

Breast cancer is one of the most common malignant tumors in women worldwide.^[1]^ About 75% of breast cancer patients are hormone receptor-positive and endocrine adjuvant therapy is usually used. Aromatase inhibitors (AIs) are the first-line drugs for endocrine therapy for postmenopausal breast cancer. Compared with tamoxifen, they can significantly reduce the absolute risk of recurrence after 10 years and improve the overall survival rate.^[2]^ American Society of Clinical Oncology clinical practice guidelines recommended that the treatment of AIs in postmenopausal women with hormone receptor-positive breast cancer was extended to 10 years, especially those with node-positive patients.^[3]^ Despite its benefits in breast cancer treatment, its musculoskeletal impact cannot be ignored. Leonor et al found that time under AIs is an independent risk factor for joint pain and bone loss, with reduced disease-free survivalin patients.^[4]^ In the IBIS-II prevention trial, patients who took anastrozole for 5 years had a significant decrease in bone mineral density.^[5]^ Nearly 10% of breast cancer patients discontinued AIs due to aromatase inhibitor-associated musculoskeletal symptoms (AIMSS).^[6]^

There are a variety of treatment methods for AIMSS, such as nonsteroidal anti-inflammatory drugs and vitamin D,^[7]^ but the efficacy is not clear. There are also exercise therapies such as Tai Chi, yoga, and Pilates to treat AIMSS, but the symptoms of bone pain and joint pain have not been significantly improved.^[8,9]^ At present, traditional Chinese medicine (TCM) has received more and more attention in the treatment of AIMSS. It is reported that Chinese herbs such as Rehmannia glutinosa, Corydalis, and Epimedium inhibit osteoclast formation, improve osteoporosis, and relieve bone pain.^[10–12]^ In clinical studies, Chinese herbal decoction relieves pain and bone loss in patients with AIMSS.^[13–15]^ However, the studies were scattered and the sample size was small. This study aimed to evaluate the efficacy and safety of TCM in the treatment of AIMSS and to provide an evidence-based basis for clinical treatment.

## 2 Methods

### 2.1 Registration

This protocol is based on the preferred reporting item for systematic review and meta-analysis protocols (PRISMA-P).^[16]^ This study is registered in the Prospero Registry of the University of York with the registration number CRD42020192553 (URL: https://www.crd.york.ac.uk/prospero/display_record.php?ID=CRD42020192553). The study used a population-based intervention comparative outcome study design framework (PICOS) to guide eligibility criteria.

### 2.2 Eligibility criteria

#### 2.2.1 Types of study

**Figure 1:**
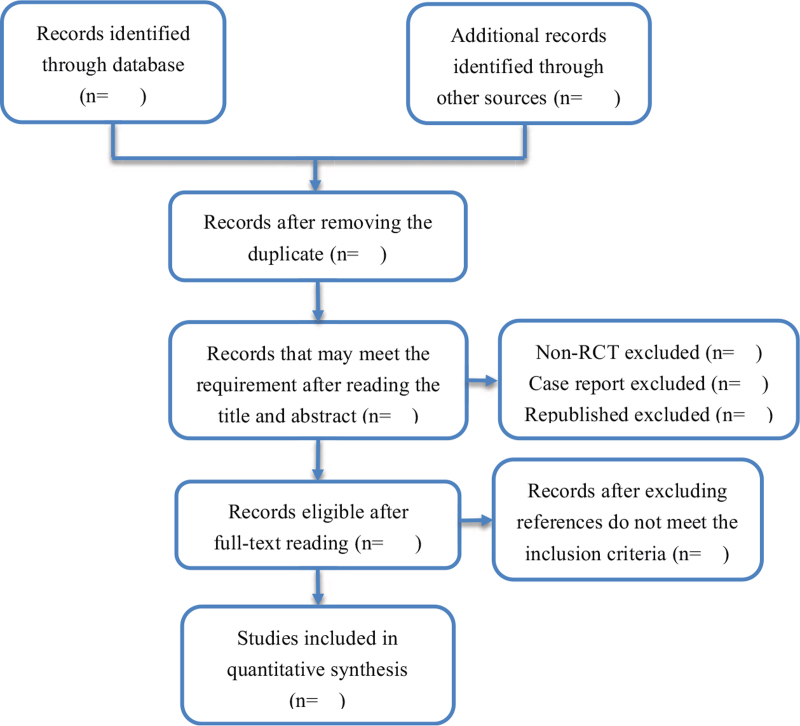
Flow diagram of studies selection process.

This review will include randomized controlled trials from inception to October 2021 in English and Chinese. Cross-sectional studies, case reports, and cohort studies are not included. Figure 1 depicts the process of studies screening.

#### 2.2.2 Types of participants

We will include studies of evaluation for pathological diagnosis of hormone receptor-positive postmenopausal breast cancer patients.

#### 2.2.3 Types of interventions

Both nondrug therapy and drug therapy will be included in the study. Aerobic exercise, yoga, Taiji, and acupuncture are nondrug therapy. Drug therapy includes analgesics, hormonal drugs, bisphosphonates, vitamin D, omega-3 fatty acids, and Chinese herbal medicine.

#### 2.2.4 Comparisons

The treatment of the control group will include placebo, blank control, routine nursing. There is no limit to the duration of treatment.

#### 2.2.5 Outcomes

Pain: Evaluating the change of joint pain degree before and after treatment against the Numerical Rating Scale, the Visual Analogue Scale, the Verbal Rating Scale (VRS), or Western Ontario and McMaster universities.^[17]^

Quality of life: The functional assessment of cancer therapy breast cancer-specific is used to evaluate the change of quality of life after treatment and at baseline.

Bone mineral density and bone metabolism markers: The indicators of bone metabolism markers include bone alkaline phosphate, osteocalcin,^[18]^ and tartrate-resistant acid phosphatase.

### 2.3 Information sources

We will search the following databases from January 2010 to October 2021: PubMed (MEDLINE), the Cochrane Library, Web of Science, the Cochrane Controlled Register of Trials (CENTRAL), Chinese National Knowledge Infrastructure (CNKI), the Chinese Science and Technology Periodical Database (VIP), Wanfang Database, and Chinese Biological Medical Database (CBM). English and Chinese studies will be included.

### 2.4 Search strategy

The search strategy of PubMed is as follows in Table 1.

**Table 1:** The search strategy of PubMed.

No.	Search terms
#1	Breast cancer[Mesh Terms]
#2	Aromatase inhibitors [Mesh Terms]
#3	#1 and #2
#4	Traditional Chinese medicine [Mesh Terms]
#5	Bisphosphonates [Mesh Terms]
#6	Tai chi [Mesh Terms]
#7	Yoga [Mesh Terms]
#8	Strength training [Mesh Terms]
#9	#4 or #5 or #6 or #7 or #8
#10	Arthritis [Mesh Terms]
#11	Osteoporosis [Mesh Terms]
#12	Bone loss [Mesh Terms]
#13	Joint pain [Mesh Terms]
#14	#10 or #11 or #12 or #13
#15	#3 and #9 and #14

### 2.5 Data collection

Two reviewers will read the abstract to screen the initial eligible studies, and then determine the final included studies through reading the full text, and finally, the 2 reviewers cross-checked the screening result. If the 2 reviewers have different opinions about the selection of studies, they can solve the problem through discussion with a third reviewer to make a decision. If the information is insufficient, we will contact the author via email for more comprehensive information. Data extraction contents include the author's name, year of publication, tumor stage, intervention measures, treatment, time, follow-up time, sample size. The flow chart is shown in Figure 1.

### 2.6 Quality of Methodological assessment

The risk assessment criteria for bias provided by Cochrane System Reviewers manual 5.1.0 will be used to evaluate the quality of the included studies, including random methods, blind method, allocation concealment, the integrity of data results, and selective reporting. Accordingly, the quality of the included literature will be divided into three: low bias (Grade A)—meet all quality standards; moderate bias (Grade B)—≥1 quality standards are partially met; high bias (Grade C)—≥1 quality standards are not met. Two reviewers will conduct quality evaluation independently and conclude the discussion with a third reviewer in case of any differences.

### 2.7 Assessment of heterogeneity

*χ*^2^ test will be used to assess the heterogeneity of the included studies, and the degree of heterogeneity will be expressed by *I*^*2*^; heterogeneity is acceptable if *I*^*2*^≤50%. If the test for heterogeneity result is *P* > .10, indicating that the homogeneity of the included studies is high, we will use the fixed-effect model for analysis. If the heterogeneity test results *P* ≤ .10, we will analyze the cause of heterogeneity and conduct a subgroup analysis. If heterogeneity exists after the above methods, a random-effect model or no meta-analysis will be selected.

### 2.8 Data analysis

The Rev Man 5.3 statistical software will be used for meta-analysis. We will assess the differences between the experimental group and the control group, and the differences between the same group before and after treatment will also be assessed. Relative risk will be used as the statistic for dichotomous data, and weighted mean difference will be used for continuous data, 95% confidence interval and *P* value are required for all analytical data.

For studies with high heterogeneity, we will look for possible sources of heterogeneity, such as intervention methods, regional differences, and so on, and conduct subgroup analysis to reduce such heterogeneity.

We will assess publication bias for outcomes with ≥10 included studies by funnel plots, try our best to reduce publication bias, excluding low-quality studies, and test the stability of the effect size estimation through sensitivity analysis. If the bias affects the system evaluation, we will report faithfully.

## 3 Discussion

Musculoskeletal symptoms such as bone pain, arthralgia, and bone loss are common adverse effects of AIs in breast cancer patients. The incidence of AI-associated joint pain or stiffness approaches 50%.^[19]^ Patients taking AIs for 5 years have a fracture rate of 18% to 20%.^[20]^ This seriously affects the patients’ quality of life, and they will stop taking AIs because of the above reasons, which will affect the treatment of breast cancer. Therefore, AIMSS are of increasing concern to physicians and patients. The most common clinical treatment for AIMSS are NSAIDs, exercise, yoga, omega-3 fatty acids, and acupuncture,^[21–23]^ but there is no clear curative effect. TCM has been widely used in clinical practice; especially, TCM decoction is very common. We designed this study to integrate the methods of TCM treatment of AIMSS and provide a reliable basis for the optimal treatment strategy of TCM treatment of AIMSS.

## Author contributions

**Conceptualization:** Meng Hui, Yu Mingwei, Wang Xiaomin

**Data curation:** Meng Hui, Chen Yuhan, Wang Xiaomin

**Formal acquisition:** Meng Hui, Chen Yuhan, Yu Mingwei

**Formal analysis:** Meng Hui, Chen Yuhan, Yu Mingwei.

**Funding acquisition:** Yu Mingwei, Wang Xiaomin

**Investigation:** Meng Hui, Chen Yuhan

**Methodology:** Meng Hui, Chen Yuhan, Yu Mingwei

**Project administration:** Meng Hui, Yu Mingwei, Wang Xiaomin

**Resources:** Meng Hui, Chen Yuhan, Yu Mingwei, Wang Xiaomin

**Software:** Meng Hui, Chen Yuhan, Yu Mingwei, Wang Xiaomin

**Supervision:** Yu Mingwei, Wang Xiaomin

**Validation:** Meng Hui, Yu Mingwei

**Visualization:** Yu Mingwei, Wang Xiaomin

**Writing – original draft:** Meng Hui, Chen Yuhan

**Writing – review & editing:** Meng Hui, Yu Mingwei, Wang Xiaomin
